# Paintable soft photonic architectures featuring multi-stable light-actuation

**DOI:** 10.1038/s41377-025-02083-7

**Published:** 2026-01-01

**Authors:** Honglong Hu, Wentan Wan, Xuan Liu, Xinshi Liang, Conglong Yuan, Yiran Ren, Yuxing Zhan, Zhi-Gang Zheng, Wei-Hong Zhu

**Affiliations:** 1 Key Laboratory for Advanced Materials and Joint International Research Laboratory of Precision Chemistry and Molecular Engineering, Shanghai Key Laboratory of Functional Materials Chemistry, Feringa Nobel Prize Scientist Joint Research Center, Institute of Fine Chemicals, Frontiers Science Center for Materiobiology and Dynamic Chemistry, School of Chemistry and Molecular Engineering, East China University of Science and Technology, Shanghai, 200237 China; 2 School of Physics, East China University of Science and Technology, Shanghai, 200237 China; 3 School of Materials Science and Engineering, East China University of Science and Technology, Shanghai, 200237 China; 4 Center of Photosensitive Chemicals Engineering, East China University of Science and Technology, Shanghai, 200237 China

**Keywords:** Liquid crystals, Photonic devices

## Abstract

Dynamic photoprogramming of paintable liquid crystal photonic devices with multi-stability shows practical application in smart soft materials and responsive optics. However, there exist three key challenges that limit their development: achieving precise paintability with controllable viscosity and resolution, maintaining well-ordered liquid crystal photonic structures, and enabling multi-stable photoresponsive behavior. Here, we address these limitations by incorporating an intrinsic photoswitch into a cellulose-based liquid crystal system, further constructing a unique paintable helical photonic architecture featuring both multi-stability and dynamic light-actuation. The intrinsic chiral photoswitch enables multi-stable modulation of helical pitch, while optimized viscosity restrains the remarkable fluidity of traditional liquid crystal systems and matches proper surface anchoring, thereby allowing for paintability and programming of a photonic device. The cutting-edge single-step painting enables highly efficient, large-area and well-defined patterning of helical architectures on diverse flexible substrates, thereby promoting prospective applications in anti-counterfeiting, information encryption, and smart window-film. This strategy establishes a robust and versatile foundation that integrates practical explorations in soft matter photonics with state-of-the-art engineering applications, such as multifunctional interactive optical information systems and advanced intelligent flexible sensors.

## Abstract (web summary)

Programmable multi-stable liquid crystal photonic devices achieve paintable patterning on flexible substrates through innovative photoswitch technology and viscosity control, offering breakthrough for anti-counterfeiting, information encryption, and smart window films.

## Graphical abstract

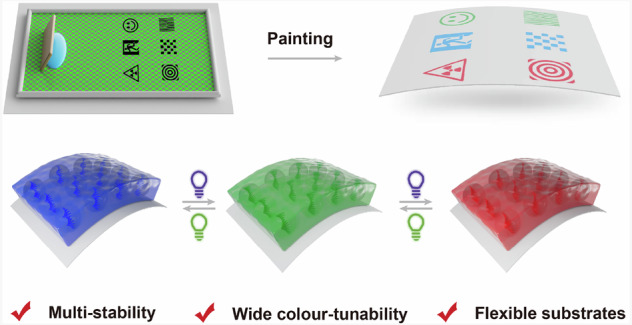

## Introduction

Light-actuated paintable liquid crystal (LC) photonic devices have been an attractive topic for years because of their great potential in the next-generation intelligent soft systems, such as smart optical and integrated systems^1–6^. However, the absence of photoresponsive multi-stable materials compatible with a straightforward and scalable painting restricts their applications, highlighting the critical challenge of achieving dynamic photomanipulation and multi-stability in such systems^7,8^. Photoresponsive cholesteric liquid crystals (CLCs) combine the precise, remote, and non-invasive^9^ control with self-organized helical superstructures^10^, have captured significant attention for their multifunctional photonic properties^11–15^, which are widespread in the fields of adaptive optical systems^16–18^, information processors^19–21^, and emissive photonic devices^22–24^. Over the years, various approaches have been proposed to modulate the helical structure of CLC using molecular photoswitches^25–28^, such as azobenzenes, spiropyrans and diarylethenes. However, current implementations of these functional CLC-based optical devices require rigid glass encapsulation to maintain surface anchoring and prevent distortion from material fluidity, fundamentally restricting their potential in flexible photonic applications. Painting represents a simple and effective approach for fabricating flexible optical devices^29–34^. Consequently, developing paintable CLC photonic architectures that simultaneously maintain well-ordered helical structures, robust photonic performance, dynamically tunable optical responses, and substrate-independent stability remains a critical challenge in advanced photonic materials.

**Fig. 1:**
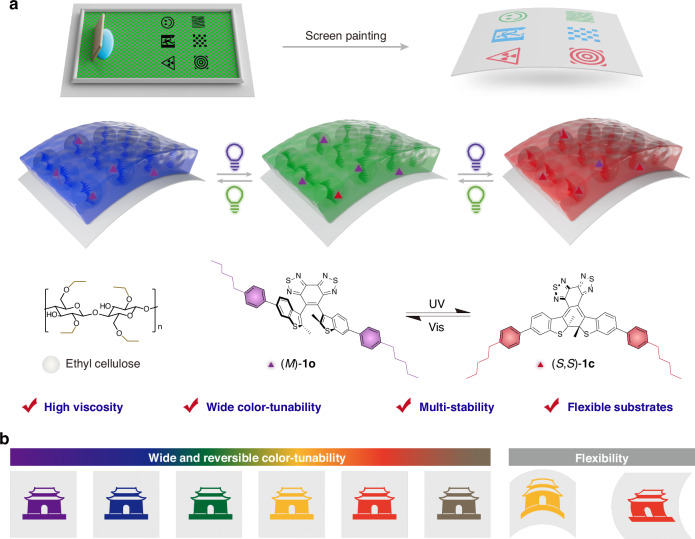
Paintable LC architectures featuring photoprogrammable multi-stable status. **a** Photonic architecture with photoprogrammable multi-stable status is constructed by painting on a flexible substrate. Here, the intrinsic chiral photoswitch induces a broad range of structural color variations due to helical pitch expansion. The introduction of ethyl cellulose increases the viscosity of the entire liquid crystal system, thereby ensuring the on-demand painting. **b** Flexible optical labels with wide and reversible color-tunability have been achieved

Here, we develop a light-actuated paintable soft photonic architecture engineered through the incorporation of ethyl cellulose as a multifunctional additive. This approach enables precise modulation of the CLC system, achieving optimal paintability while maintaining robust surface anchoring strength and well-defined optical patterns across diverse substrates. A unique intrinsic chiral photoswitch with excellent thermo-stability is introduced into LC to enable remarkable manipulation of helical pitch, thereby enabling precise multi-stable modulation across a wide spectral bandwidth spanning from the entire visible to the near-infrared region upon light stimulation (Fig. 1a). Notably, these optical architectures enable large-area, scalable and accurate painting while simultaneously allowing for on-demand photoprogrammable micropatterns, significantly advancing their potential applications towards flexible and multi-stable optics, especially in anti-counterfeiting, information encryption, and smart window-film (Fig. 1b). This study puts forward transformative practical horizons for multifunctional interactive optical information systems and advanced intelligent flexible sensors.

## Results

### Paintable soft photonic architectures: high viscosity and multi-stability

**Fig. 2:**
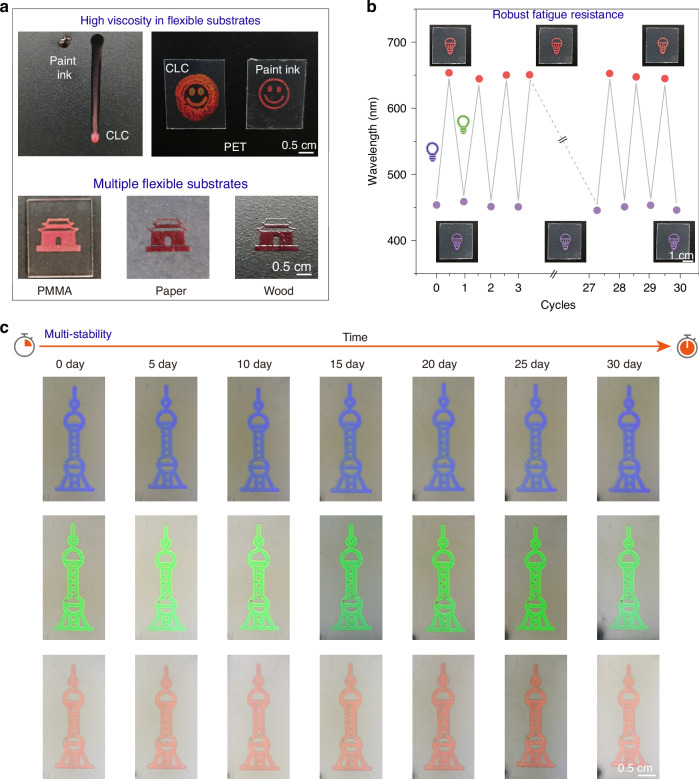
Paintable photonic architectures: high viscosity on diverse substrates, robust fatigue resistance, and multi-stability. **a** Compare to pure CLC, the paintable ink shows high viscosity without obvious fluidity on the vertical substrate, and the clear pattern can be easily painted on multiple flexible substrates. **b** Paintable photonic architectures can be switched between purple and red 30 cycles while still maintaining clear colors and edges under alternating exposure to UV and visible light. **c** Paintable photonic architectures exhibit outstanding thermal stability with unaffected color and functionality after 30 days

To achieve a wide range of structural color controlled by light, we strategically incorporated the intrinsically chiral diarylethene derivative developed by our group^28^ as the fundamental building block for constructing the CLC. This photoswitch possessed three critical features: avoidance of multi-centered chirality, significant helical pitch modulation, and robust thermal stability, thereby enabling highly efficient optical modulation with wide-range pitch tuning in CLC systems. Moreover, the photoswitch exhibits excellent bistability across all intermediate configurations during light-driven interconversion between its open (*M*)-**1o** and closed (*S*,*S*)-**1c** states, enabling reliable multi-stable functionality. However, this CLC system exhibits inadequate viscosity, showing obvious flow traces on vertical substrates and poorly resolved pattern boundaries (Fig. 2a, Fig. S1 and Supplementary Video 1). To address these limitations, we developed a viscosity-tuning strategy through the controlled incorporation of ethyl cellulose into the CLC matrix. The CLC-to-ethyl cellulose mass ratio significantly influences optical modulation performance. A 1:1 ratio achieved a balance between the desired optical tunability and optimal viscosity (Table S1). Compositions with CLC excess suffered from poor film uniformity due to high fluidity, whereas ethyl cellulose-rich mixtures exhibited dramatically reduced optical responsiveness, either narrowing the dynamic range or eliminating tunability entirely. Furthermore, the entire material system exhibited robust interfacial interactions, as evidenced by optimal wetting/spreading characteristics across all formulated compositions (Fig. S2). The optimized system demonstrates excellent paintability, exhibiting minimal flow on vertical substrates while preserving sharp pattern definition, indicative of an ideal viscosity for precise patterning. Remarkably, this technique achieved high-definition patterning across diverse materials, including PET, PMMA, paper, and wood, highlighting its excellent compatibility for paintable photonics.

Stability and fatigue resistance represent critical performance metrics that fundamentally determine the operational reliability and long-term durability of functional devices in practical engineering applications. They are crucial for ensuring the performance, reliability, and lifetime of products. Here, the flexible photonic architecture exhibited robust fatigue resistance, maintaining almost invariable reflection color between purple and red via monitoring at least 30 switching cycles upon alternating UV and visible-light irradiation, thereby minimizing the fatigue damage and extending device life (Fig. 2b). Furthermore, they also possessed excellent thermal stability, remaining unaffected color and properties after 30 days in different temperatures (Fig. 2c, Fig. S3 and Fig. S4). These characteristics of the flexible photonic architecture established a strong foundation for their future applications, particularly in the realm of anti-counterfeiting labels and information encryption.

### Paintable soft photonic architectures: wide and reversible color-tunability

**Fig. 3:**
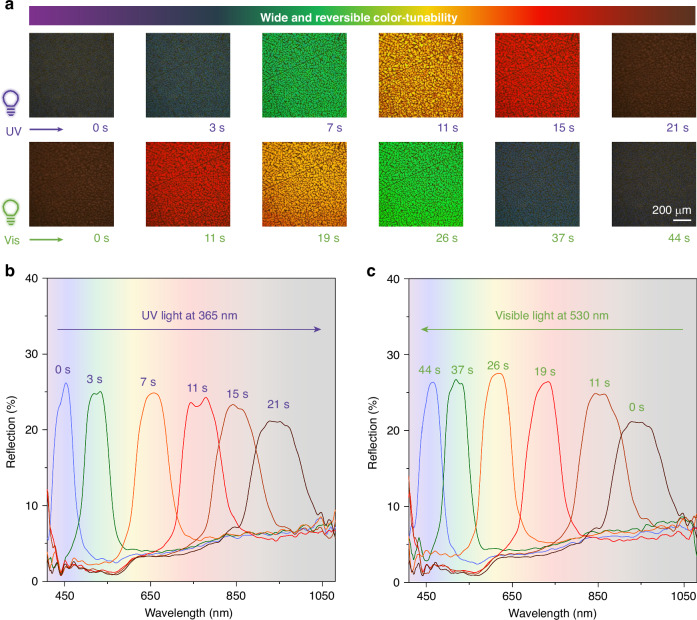
Paintable photonic superstructures with wide and reversible color-tunability. **a** Reflection color is manipulated reversibly between purple and dark red upon alternating UV- and visible-light irradiation. **b** Corresponding reflection spectra showed wide tunability from 450 nm to the near-infrared band at around 950 nm upon UV light irradiation for 21 s, and the reversible process was shown in **c** with visible light irradiation for 44 s. Here, the irradiation intensities of UV light at 365 nm and visible light at 530 nm are 4.0 mW cm^−2^

Remarkably, the developed system enables not only substrate-independent patterning capabilities but also exhibits broad-spectrum and fully reversible optical tunability using light irradiation. Here, polarized optical microscopy (POM) was employed to capture the texture images of the paintable photonic architecture with a thickness of 32.8 μm (Fig. S5). The oily texture exhibited a remarkable transition of reflection colors, progressing from an initial dark purple to blue, green, yellow, red, and eventually reaching a photostationary state with dark red upon UV light (365 nm) irradiation for 21 s, allowing for a remarkable manipulation of helical pitch. Subsequent full recovery could be achieved through irradiation with visible light (530 nm) for 44 s (Fig. 3a and Supplementary Video 2). It is important to highlight that the excellent thermal stability of photoswitches shows the stable intermediate states with desired reflection colors after removing the light. Moreover, thanks to the high viscosity of this system, we successfully captured and characterized the dynamic evolution of photoresponsive helical pitch modulation. By carefully adjusting the chiral dopant concentration to achieve micron-scale pitch dimensions, we were able to directly observe the dynamic pitch modulation in the cross-sectional view under microscopy (Fig. S6 and Supplementary Video 3).

To quantitatively characterize the reflection wavelength of the paintable photonic architecture, we employed a fiber-optic-coupled spectrometer synchronized with POM for simultaneous structural and optical analysis. The CLC-based photonic architecture exhibited excellent spectral tunability, with dynamically manipulable reflection bands spanning from 450 nm (visible) to 950 nm (near-infrared) (Fig. 3b). And the spectral could also be reversibly manipulated upon visible light irradiation (Fig. 3c). Moreover, the precise spectral modulation without obvious spectral distortion and broadband demonstrated the inherent uniform arrangement of the paintable CLC photonic superstructure. In fact, the chiral pitch transformation is critically dependent on the total irradiation energy (i.e., the product of light power and exposure duration). The necessary irradiation time follows an inverse proportionality with light power, with 365 nm irradiation inducing the target pitch significantly faster than 532 nm at equivalent power due to wavelength-dependent quantum efficiency (Fig. S7). The combination of broad spectral tunability and complete reversibility in these photoprogrammable CLCs established them as a robust material platform for responsive photonics, with immediate applications in optical sensors, displays, imaging and so on.

### Paintable micropatterns with distinct optical diffraction

**Fig. 4:**
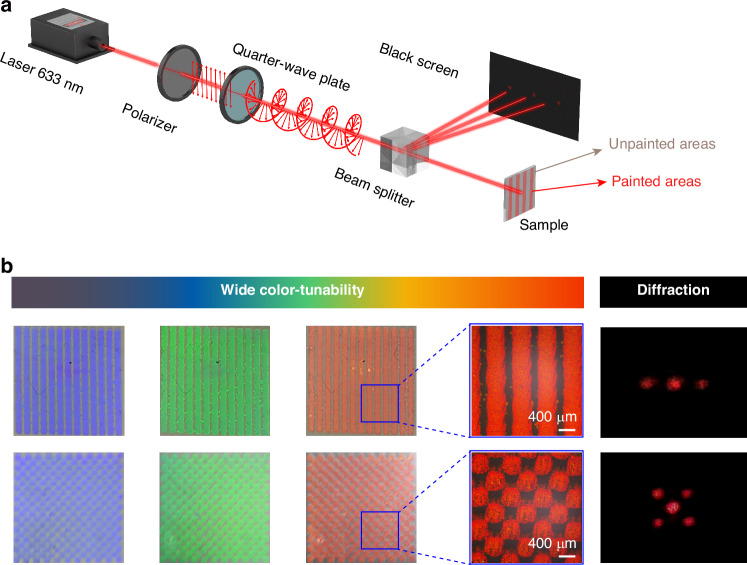
Dynamic photoprogramming of paintable micropatterns with distinct optical diffraction. **a** By using a polarizer and a quarter-wave plate, a 633 nm laser was transformed into LCP light to detect the typical diffraction patterns. **b** Photoprogrammable micropatterns and their diffraction of one-dimensional grating, fork-shaped grating, two-dimensional grating, and periodic concentric circles with dark purple, blue, green, yellow, and red reflection colors were generated using a 365 nm UV light

Distinguished from traditional LC micropatterning techniques^35–39^, our developed LC system enabled the direct fabrication of micropatterns through a single-step paintable process of photoprogrammable ink onto flexible substrates, especially for expanding the potential applications of flexible LC-based devices in emerging technological fields. To comprehensively investigate the capability of such photoprogrammable LC architecture for optics, we constructed a series of precisely controlled micropatterns capable of generating distinct optical diffraction through light manipulation (Fig. 4a). Through a silk screen featuring one-dimensional grating, fork-shaped grating, two-dimensional grating, and periodic concentric circles, four optical micropatterns were painted on PET film successively (Fig. 4b and Fig. S8). These samples initially exhibited a purple structural color, underwent sequential color transitions through blue, green, and yellow, and ultimately stabilized at a photostationary state of red upon UV light irradiation. The complete recovery could be attained by visible light irradiation. Excitingly, these micropatterns exhibited sharp boundaries and stability throughout the entire transformation process, with a demonstrated painting resolution of approximately 400 μm (Fig. S9 and Fig. S10), positioning them as promising candidates for advanced flexible photonic and optoelectronic device fabrication.

Here, we used a 633 nm laser to study the optical properties of these micropatterns, which minimally affected the photosensitive system. When the wavelength of incident light matched the selective reflection band of the patterned CLC architecture, distinct diffraction occurred due to Bragg reflection. Specifically, the 633 nm left-handed circularly polarized (LCP) light met the Bragg condition in helical CLC domains, while adjacent unstructured regions remained transparent (Fig. 4a and Fig. S11). This optical contrast at the interface between painted and unpainted regions enabled effective spatial amplitude modulation. The Bragg reflection wavelength was precisely tuned to 633 nm through controlled UV irradiation, achieving optimal diffraction efficiency in the micropatterns. Horizontally-distributed, doughnut-shaped, checkerboard-distributed, and circle-shaped diffraction spots gradually appeared on the receiving screen (Fig. 4b), further confirming the excellent stability and sharp edges of these paintable micropatterns.

### Multiple anti-counterfeiting labels

**Fig. 5:**
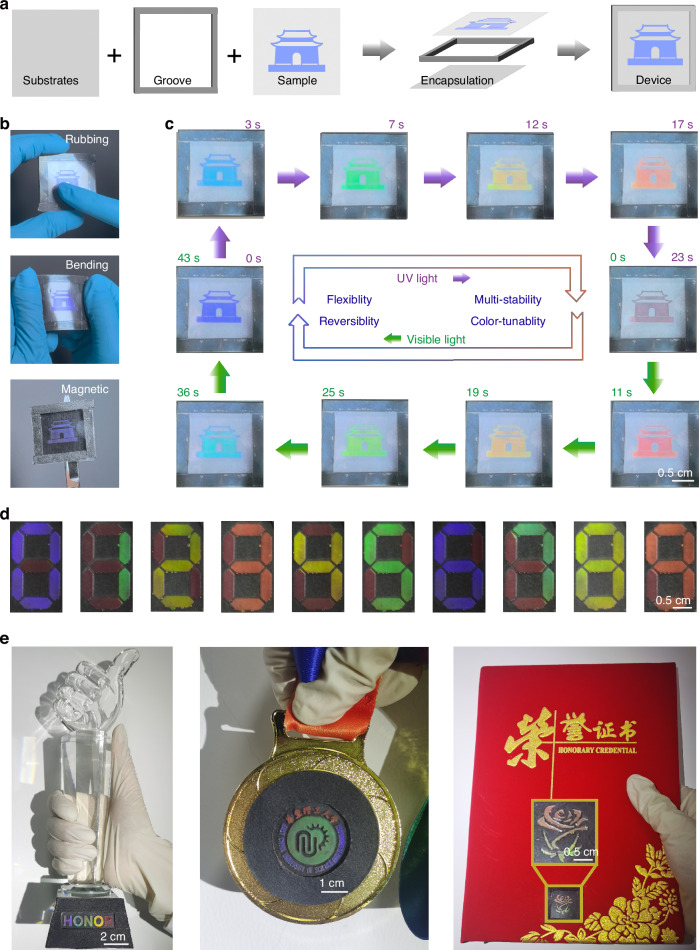
Multiple anti-counterfeiting labels with flexibility, reversibility, multi-stability and color-tunability. **a** Schematic illustration of the groove-embedded encapsulation technique. **b** Flexibility of the optical pattern on the PET film with rubbing, bending and magnetic. **c** With alternating irradiation with UV light (365 nm) and visible light (530 nm), the flexibility, multi-stability, reversibility, and color-tunability of the film are achieved. Here, the irradiation intensities of UV light at 365 nm and visible light at 530 nm are 4.0 mW cm^−2^, respectively. **d** Colorful display device of 0-9 numbers is achieved by irradiating different regions. **e** Colorful “HONOR” on the trophy, “ECUST logo” on the medal, and “rose” on the certificates are achieved

Design and fabrication of advanced security labels in a convenient way, capable of high-level security, low-cost, and large-area, have garnered significant scientific interest. The key to advanced encryption and anti-counterfeiting strategies is the storage of security elements that can be selectively and accurately modulated by specific stimuli^40–43^. In light-stimulated color-changing flexible anti-counterfeiting devices, the traditional variations in pigment or structural color are relatively limited, typically exhibiting only two- or three-color changes. Developing optical anti-counterfeiting devices with a wider range of color change can significantly increase the anti-counterfeiting security level and capacity. As a proof of concept, we achieved single-step direct writing of a “house” pattern on PET film using a paintable CLC system, successfully combining groove-embedded encapsulation with magnetic substrate integration (Fig. 5a). The optical patterns remained stable with nearly unchanged reflection spectra even after repeated rubbing and bending, while maintaining good adhesion to iron surfaces (Fig. 5b, Fig. S12 and Supplementary Video 4). Furthermore, systematic evaluation under varying pressure and pH conditions confirmed the robustness and reliability of our encapsulation approach (Fig. S13). The pattern performed a distinct purple, followed by a successive color change to blue (3 s), green (7 s), yellow (12 s), red (17 s), and dark red (23 s) with sustained UV exposure, and it recovered again via visible-light irradiation at 530 nm (Fig. 5c and Supplementary Video 5). Remarkably, owing to its excellent thermal stability, the image at any intermediate state remained unchanged, without observable color degradation or boundary diffusion, demonstrating the intrinsic multi-stable characteristics of the system. Even after 180 days, the entire transition process remained fully reversible, further confirming the long-term stability of the material (Supplementary Video 6).

For promising applications, we have designed a type of digital color display device, which can display 0-9 numbers with distinguished colors by precisely controlling the localized irradiation time (Fig. 5d and Fig. S14). This display reduced power consumption by sequentially displaying primary colors, thereby enhancing image clarity. Following the same aforementioned methodology, we generated a colorful “HONOR” on the trophy, “ECUST logo” on the medal, and “rose” on the certificates (Fig. 5e). These patterns could be reversibly displayed and encrypted through control of the light-exposed area (Figs. S15–S17). Significantly, the paintable optical architecture allowed for diverse platforms ranging from flexible wearable substrates to large-scale surface patterning, thus achieving rapid processability and high adaptability (Figs. S18 and S19 and Supplementary Video 7).

### Multifunctional smart window-film

**Fig. 6:**
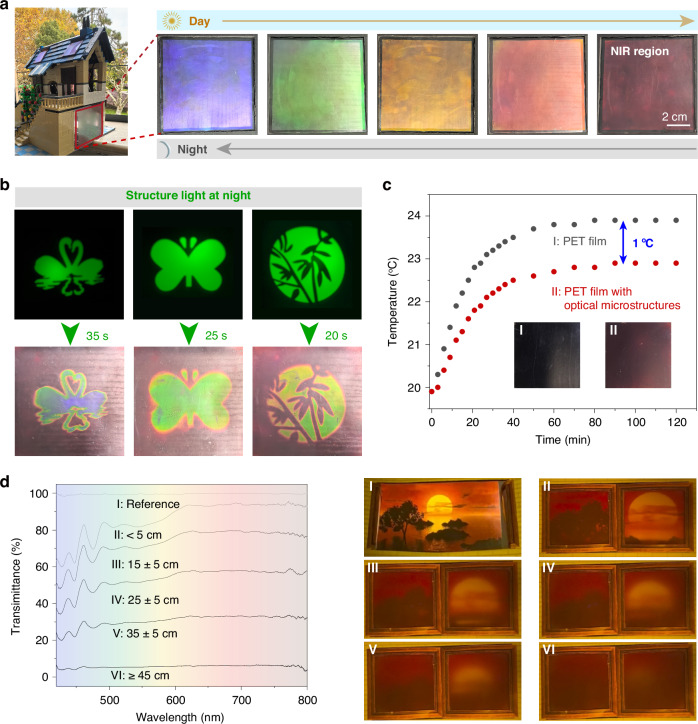
Multifunctional smart window-film. **a** Color-adaptability: structural color gradually redshifts to the near-infrared region with sunlight irradiation and reversibly returns under indoor white light at night. **b** Light-interactivity: by illuminating with structured light of various patterns, different colors and designs can be formed on the film, enhancing the interaction between the film and the user. **c** Heat-resistibility: this photoprogrammable structural color system provides effective daytime thermal management by selectively reflecting near-infrared (NIR) radiation, effectively reducing the indoor heating rate and temperature. **d** Privacy-controllability: the optical transmittance of the painted CLC system exhibits distance-dependent modulation, enabled by controlled scattering effects. The system achieves near-zero transmittance at distances beyond 45 cm for enhanced privacy, while maintaining high transparency (∼90% transmittance) within 5 cm for clear outdoor visibility

Smart window technology not only enhances the comfort and convenience of living environments but also plays a significant role in energy savings, privacy protection, and esthetics^44,45^. Thanks to the wide and reversible color tunability of this paintable photonic architecture, we have developed a multifunctional smart window-film that provides a sustainable solution by integrating color-adaptability, light-interactivity, heat-resistibility, and privacy-controllability. Depending on the intensity of UV radiation in sunlight, the window film exhibited different structural colors, ranging across the visible spectrum and even extending into the near-infrared (NIR) range. Under solar exposure, the structural color gradually redshifted toward longer wavelengths. In contrast, under indoor lighting in the evening, the film underwent a reversible blueshift, restoring its structural color to the visible spectral range (Fig. 6a and Supplementary Video 8).

Additionally, the smart film allowed for the creation of decorative motifs like swans, butterflies, and leaves through a controlled pattern-shading structure. These vivid patterns demonstrated dynamic light interaction, exhibiting structural colors that transition across the visible spectrum while preserving their intrinsic photonic properties. This unique characteristic allowed artistic designs to integrate with functional solar regulation (Fig. 6b). It is worth noting that the near-infrared structural color under sunlight exposure during the day could effectively block heat. Through experimental comparison, it had been observed that the heating rate of PET surfaces painted with photonic architecture was significantly lower, and the temperature decreased nearly 1 °C compared to pure PET surfaces (Fig. 6c and Fig. S20), which effectively reduced the energy consumption. The self-adaptive behavior of our smart window film is evident in its dynamic spectral response, where the reflection peak precisely tracks the near-infrared region under increasing light intensity (Fig. S21), demonstrating real-time photonic regulation.

Leveraging the unique light-scattering behavior of the CLC, the film created an intriguing mist-like effect. When viewed beyond 45 cm, the transmittance sharply declined to near-zero levels, effectively obscuring visibility and ensuring robust privacy. Conversely, as the observer moved within 5 cm, the transmittance rose to approximately 90%, restoring a clear view of the surroundings. This dynamic optical response enables transitions between privacy and transparency, making it ideal for adaptive smart windows or privacy-sensitive applications (Fig. 6d).

## Discussion

Light-actuated paintable LC photonic devices face fundamental challenges of achieving precise paintability with controlled viscosity and resolution, maintaining well-ordered photonic structures, and enabling robust multi-stable photoresponsiveness. This work establishes a versatile material and fabrication platform for the first time, enabling single-step painting of LC soft helical architecture with excellent photonic performances on diverse substrates, along with simultaneously achieving programmable multi-stable light-actuation. The distinctive intrinsic chiral photoswitch with excellent thermo-stability can facilitate a multi-stable chiral LC system capable of digitally photoprogramming on structural colors across the entire visible to near-infrared spectral range (up to 600 nm). These chiral photonic architectures were transferred onto diverse flexible substrates through a viscosity-engineered single-step painting approach utilizing ethyl cellulose, achieving customizable painting of whatever macro- and micro-patterns. Leveraging these characteristics, we have expanded the scope of photonic applications, such as anti-counterfeiting, information encryption, and smart window-film, solidifying the versatility and transformative potential in the future emerging photonics technologies. Such paintable LC architectures harnessing photoprogrammable multi-stability provide a robust foundation for developing smart photosensitive materials in soft matter photonics, marking paradigm-transforming scientific contributions that drive disruptive progress in the field.

## Materials and methods

### Materials

All commercially available starting reagents and solvents were used directly without further treatment unless otherwise specified. The photoresponsive chiral molecular switches (*M*)-**1o** were prepared according to the established methods^28^. Commercially available *α*-Terpineol (J&K Scientific, China), Ethyl cellulose (J&K Scientific, China), chiral dopant S5011 (Merck, Germany), and the commercial LC TEB300 (Slichem, China) were purchased for preparing the paintable CLC system.

### Preparation of the paintable CLC system

Our programmable photonic system consists of two fundamental components that work synergistically to enable the creation of paintable optical superstructures. The first component involves the preparation of a photoresponsive CLC system. The photoswitch (*M*)-**1o** (2.4 wt%) and chiral molecule S5011 (1.2 wt%) were homogeneously mixed with commercial LC (TEB300). For the second component, ethyl cellulose (1 g) was dissolved in *α*-terpineol (1 mL) to prepare a paintable ink. To fabricate the final paintable optical superstructures, the photoprogrammable CLC system and the ethyl cellulose-based ink were thoroughly mixed in a 1:1 weight ratio. The resulting composite was then deposited onto target substrates via screen painting, enabling precise patterning and large-area fabrication.

### Characterization of the CLC superstructures

All photochromic reactions of CLC films were irradiated by a 530 nm collimated LED light source (M530L3, 4.0 mW cm^−2^, Thorlabs) or a 365 nm UV LED source (SunSpot 2, 4.0 mW cm^−2^, Uvitron). The optical textures of LC samples were observed by a polarized optical microscope (LVPOL 100, Nikon) with crossed polarizers under reflection mode and were recorded using a charge-coupled device (CCD) camera. Generally, the fiber-coupled spectrometer (Avaspec-ULS2048, resolution: 2 nm, 200–1100 nm) was used to detect the reflection spectra. The optical diffraction technique was characterized using 633 nm He–Ne lasers.

## Supplementary information

Supplementary Information

Viscosity of the paintable photonic architecture

Reflection color change of paintable photonic architecture

Photoprogramming cross-sectional helical pitch

Flexible label of “house”

Multi-color label of “house”

Multi-color label of “house” after 180 days

Wearable multi-color device

Multifunctional smart window film

## Data Availability

The data that support the findings of this study are available from the corresponding author upon reasonable request.

## References

[1] Fang, Z. Z. et al. 3D printable elastomers with exceptional strength and toughness. *Nature* **631**, 783–788 (2024). 38961297 10.1038/s41586-024-07588-6

[2] Zhang, W. et al. Printing of 3D photonic crystals in titania with complete bandgap across the visible spectrum. *Nat. Nanotechnol.* **19**, 1813–1820 (2024). 39251863 10.1038/s41565-024-01780-5

[3] Choi, S. et al. Structural color printing via polymer-assisted photochemical deposition. *Light* **11**, 84 (2022). 10.1038/s41377-022-00776-x PMC8986859 35387968

[4] Kamal, W. et al. On-demand pitch tuning of printed chiral nematic liquid crystal droplets. *Mater. Today Adv.* **19**, 100416 (2023).

[5] Zhao, J. Y. et al. Full-color laser displays based on organic printed microlaser arrays. *Nat. Commun.* **10**, 870 (2019). 30787345 10.1038/s41467-019-08834-6 PMC6382787

[6] Yang, Y. Z. et al. High-throughput printing of customized structural-color graphics with circularly polarized reflection and mechanochromic response. *Matter* **7**, 2091–2107 (2024).

[7] Lugger, S. J. D. et al. Hydrogen-bonded supramolecular liquid crystal polymers: smart materials with stimuli-responsive, self-healing, and recyclable properties. *Chem. Rev.* **122**, 4946–4975 (2022). 34428022 10.1021/acs.chemrev.1c00330 PMC8915167

[8] del Pozo, M. et al. 4D printing of liquid crystals: what’s right for me?. *Adv. Mater.* **34**, 2104390 (2022). 10.1002/adma.202104390 34716625

[9] Irie, M. et al. Photochromism of diarylethene molecules and crystals: memories, switches, and actuators. *Chem. Rev.* **114**, 12174–12277 (2014). 25514509 10.1021/cr500249p

[10] Bisoyi, H. K. & Li, Q. Light-driven liquid crystalline materials: from photo-induced phase transitions and property modulations to applications. *Chem. Rev.* **116**, 15089–15166 (2016). 27936632 10.1021/acs.chemrev.6b00415

[11] Bisoyi, H. K. & Li, Q. Liquid crystals: versatile self-organized smart soft materials. *Chem. Rev.* **122**, 4887–4926 (2022). 34941251 10.1021/acs.chemrev.1c00761

[12] Liu, X. et al. Programming dual-color circularly polarized luminescence with self-organized soft photonic helix. *Laser Photonics Rev.* **18**, 2300603 (2024).

[13] Yin, K. et al. Advanced liquid crystal devices for augmented reality and virtual reality displays: principles and applications. *Light* **11**, 161 (2022). 10.1038/s41377-022-00851-3 PMC9151772 35637183

[14] Huang, Y. L. et al. Photocontrollable elongation actuation of liquid crystal elastomer films with well-defined crease structures. *Adv. Mater.* **35**, 2304378 (2023). 10.1002/adma.202304378 37421658

[15] Hu, H. L. et al. Multiple degrees of freedom photoprogramming of soft helical microstructures featuring copper-gated photoswitch. *Matter* **6**, 3927–3939 (2023).

[16] Li, P. et al. Wearable and interactive multicolored photochromic fiber display. *Light* **13**, 48 (2024). 10.1038/s41377-024-01383-8 PMC10866970 38355692

[17] Qian, N. N. et al. Patterned photonic actuators with dynamic shape-morphing and color-changing capabilities fabricated by athermal embossing technology. *Angew. Chem. Int. Ed.* **63**, e202406534 (2024). 10.1002/anie.202406534 38693606

[18] Long, G. Y. et al. Photoresponsive biomimetic functions by light-driven molecular motors in three dimensionally printed liquid crystal elastomers. *J. Am. Chem. Soc.* **146**, 13894–13902 (2024). 38728606 10.1021/jacs.4c01642 PMC11117400

[19] Qin, L. et al. Piecewise phototuning of self-organized helical superstructures. *Adv. Mater.* **30**, 1704941 (2018). 10.1002/adma.201704941 29265677

[20] Liu, J. L. et al. Visible-light-programmed patterning in dynamically bonded cholesteric liquid crystal elastomer. *Nat. Commun.* **15**, 10367 (2024). 39609449 10.1038/s41467-024-54881-z PMC11604966

[21] Hebner, T. S. et al. Shape permanence in diarylethene-functionalized liquid-crystal elastomers facilitated by thiol-anhydride dynamic chemistry. *Angew. Chem. Int. Ed.* **61**, e202116522 (2022). 10.1002/anie.202116522 35023253

[22] Hu, H. L. et al. A quadri-dimensional manipulable laser with an intrinsic chiral photoswitch. *Adv. Mater.* **34**, 2110170 (2022). 10.1002/adma.202110170 35143699

[23] Wu, Y. et al. Liquid crystal assembly for ultra-dissymmetric circularly polarized luminescence and beyond. *J. Am. Chem. Soc.* **145**, 12951–12966 (2023). 37276078 10.1021/jacs.3c01122

[24] Zhang, J. Y. et al. Programmable dynamic information storage composite film with highly sensitive thermochromism and gradually adjustable fluorescence. *Adv. Mater.* **36**, 2305872 (2024). 10.1002/adma.202305872 38016803

[25] Lin, S. Y. et al. Fluorescent photochromic α-cyanodiarylethene molecular switches: an emerging and promising class of functional diarylethene. *Adv. Funct. Mater.* **31**, 2007957 (2021).

[26] Pang, X. L. et al. Photodeformable azobenzene-containing liquid crystal polymers and soft actuators. *Adv. Mater.* **31**, 1904224 (2019). 10.1002/adma.201904224 31595576

[27] Bisoyi, H. K. & Li, Q. Light-directing chiral liquid crystal nanostructures: from 1D to 3D. *Acc. Chem. Res.* **47**, 3184–3195 (2014). 25181560 10.1021/ar500249k

[28] Zheng, Z. G. et al. Digital photoprogramming of liquid-crystal superstructures featuring intrinsic chiral photoswitches. *Nat. Photonics* **16**, 226–234 (2022).

[29] Zheng, Z. G. et al. Wide tunable lasing in photoresponsive chiral liquid crystal emulsion. *J. Mater. Chem. C* **3**, 2462–2470 (2015).

[30] Fu, Y. et al. Reversible photochromic photonic crystal device with dual structural colors. *ACS Appl. Mater. Interfaces* **14**, 29070–29076 (2022). 35666620 10.1021/acsami.2c03771

[31] Li, R. J. et al. Dynamic high-capacity structural-color encryption via inkjet printing and image recognition. *Adv. Funct. Mater.* **34**, 2404706 (2024).

[32] Chen, M. et al. 4D printing of reprogrammable liquid crystal elastomers with synergistic photochromism and photoactuation. *Adv. Mater.* **36**, 2303969 (2024). 10.1002/adma.202303969 37432879

[33] Li, X. H. et al. Cholesteric liquid crystal elastomer coatings with brilliant structural colors and mechanochromic response fabricated by spray deposition. *Adv. Funct. Mater.* **35**, 2412298 (2025).

[34] Yang, X. et al. Robust integration of polymerizable perovskite quantum dots with responsive polymers enables 4D-printed self-deployable information display. *Matter* **6**, 1278–1294 (2023).

[35] Feng, Z. Y. et al. Dynamic multimodal information encryption combining programmable structural coloration and switchable circularly polarized luminescence. *Nat. Commun.* **16**, 2264 (2025). 40050269 10.1038/s41467-025-57649-1 PMC11885572

[36] Wang, D. et al. Color liquid crystal grating based color holographic 3D display system with large viewing angle. *Light* **13**, 16 (2024). 10.1038/s41377-023-01375-0 PMC10788332 38221521

[37] Ma, L. L. et al. Self-assembled liquid crystal architectures for soft matter photonics. *Light* **11**, 270 (2022). 10.1038/s41377-022-00930-5 PMC9470592 36100592

[38] Zhang, R. C. et al. Advanced liquid crystal-based switchable optical devices for light protection applications: principles and strategies. *Light* **12**, 11 (2023). 10.1038/s41377-022-01032-y PMC9807646 36593244

[39] Ma, J. et al. Liquid crystals for advanced smart devices with microwave and millimeter-wave applications: recent progress for next-generation communications. *Adv. Mater.* **35**, 2302474 (2023). 10.1002/adma.202302474 37225649

[40] Guo, Q. et al. Multimodal-responsive circularly polarized luminescence security materials. *J. Am. Chem. Soc.* **145**, 4246–4253 (2023). 10.1021/jacs.2c13108 36724236

[41] Zhang, X. et al. Liquid crystal-templated chiral nanomaterials: from chiral plasmonics to circularly polarized luminescence. *Light* **11**, 223 (2022). 10.1038/s41377-022-00913-6 PMC9283403 35835737

[42] Qin, L. et al. Geminate labels programmed by two-tone microdroplets combining structural and fluorescent color. *Nat. Commun.* **12**, 699 (2021). 33514695 10.1038/s41467-021-20908-y PMC7846849

[43] Lin, S. Y. et al. Photo-triggered full-color circularly polarized luminescence based on photonic capsules for multilevel information encryption. *Nat. Commun.* **14**, 3005 (2023). 37231049 10.1038/s41467-023-38801-1 PMC10212932

[44] Huang, Z. K. et al. An efficient and flexible bifunctional dual-band electrochromic device integrating with energy storage. *Nano-Micro Lett.* **17**, 98 (2025). 10.1007/s40820-024-01604-0 PMC11680540 39729147

[45] Kamal, W. et al. Spatially patterned polymer dispersed liquid crystals for image-integrated smart windows. *Adv. Opt. Mater.* **10**, 2101748 (2022).

