# Supplementary material for: Paintable soft photonic architectures featuring multi-stable light-actuation
Source: Light Sci Appl. 2026 Jan 1;15:10. doi: 10.1038/s41377-025-02083-7 (PMC12756290; doi:10.1038/s41377-025-02083-7)
Supplement: Supplementary file 1 — Supplementary Information [file 41377_2025_2083_MOESM1_ESM.pdf]

Supplementary Information

Paintable soft photonic architectures featuring multi-stable light-actuation

Honglong Hu[1,2], Wentan Wan[3], Xuan Liu[3], Xinshi Liang[1], Conglong Yuan[1,2], Yiran Ren[2], Yuxing Zhan[3], Zhi-Gang Zheng[2,3,*] & Wei-Hong Zhu[1,4,*]

[1]Key Laboratory for Advanced Materials and Joint International Research Laboratory of Precision Chemistry and Molecular Engineering, Shanghai Key Laboratory of Functional Materials Chemistry, Feringa Nobel Prize Scientist Joint Research Center, Institute of Fine Chemicals, Frontiers Science Center for Materiobiology and Dynamic Chemistry, School of Chemistry and Molecular Engineering, East China University of Science and Technology, Shanghai 200237, China.

[2]School of Physics, East China University of Science and Technology, Shanghai 200237, China.

[3]School of Materials Science and Engineering, East China University of Science and Technology, Shanghai 200237, China.

[4]Center of Photosensitive Chemicals Engineering, East China University of Science and Technology, Shanghai 200237, China.

*Correspondence: Zhi-Gang Zheng (zgzheng@ecust.edu.cn) or Wei-Hong Zhu (whzhu@ecust.edu.cn)

This file includes:

Supplementary Figures 1-21

Supplementary Table 1

Other Supplementary Materials for this manuscript include the following:

Supplementary Video 1. Viscosity of the paintable photonic architecture.

Supplementary Video 2. Reflection color change of paintable photonic architecture.

Supplementary Video 3. Photoprogramming cross-sectional helical pitch.

Supplementary Video 4. Flexible label of “house”.

Supplementary Video 5. Multi-color label of “house”.

Supplementary Video 6. Multi-color label of “house” after 180 days.

Supplementary Video 7. Wearable multi-color device.

Supplementary Video 8. Multifunctional smart window-film.

Supplementary Figures and Table

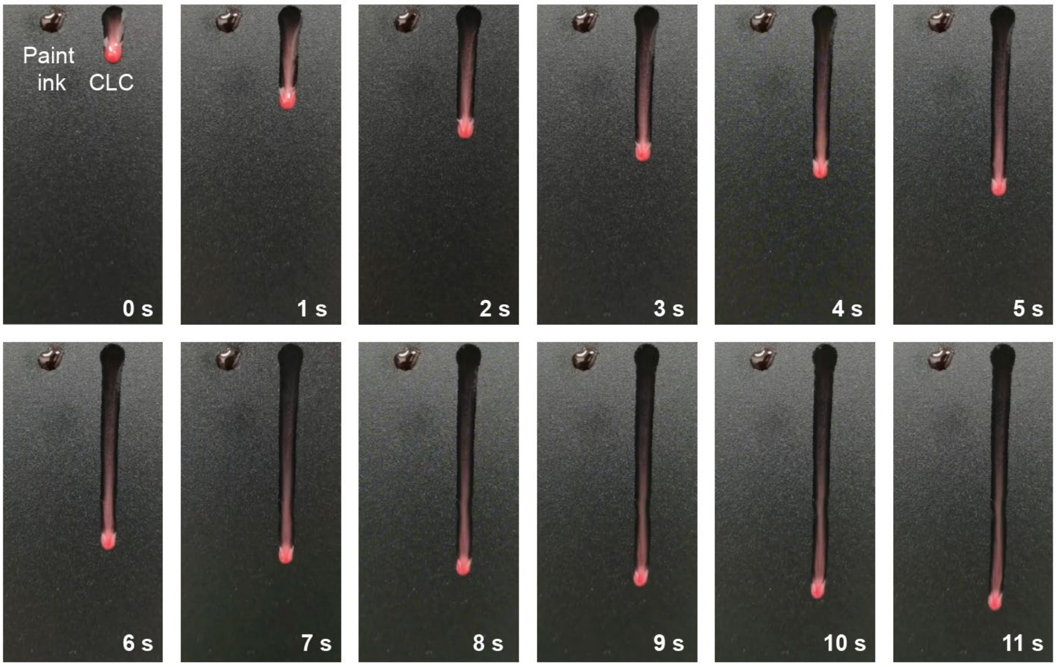

Fig. S1 Comparison of the fluidity of paint ink and CLC at different time points on a vertical substrate.

Table S1 Optimizing paintable CLC system by introducing ethyl cellulose.

M1 (g L^{-1})	M1 : M2	Tunability (nm)	Viscosity (cP)
500	0.5 : 0.5	480 ± 20	919 ± 10
800	0.5 : 0.5	480 ± 20	4730 ± 50
1000	0.5 : 0.5	480 ± 20	14600 ± 150
1000	0.6 : 0.4	350 ± 20	19800 ± 200
1000	0.7 : 0.3	20 ± 20	24600 ± 250

Note: M1: Ethyl cellulose, M2: CLC system (including (*M*)-**1o** in TEB300)

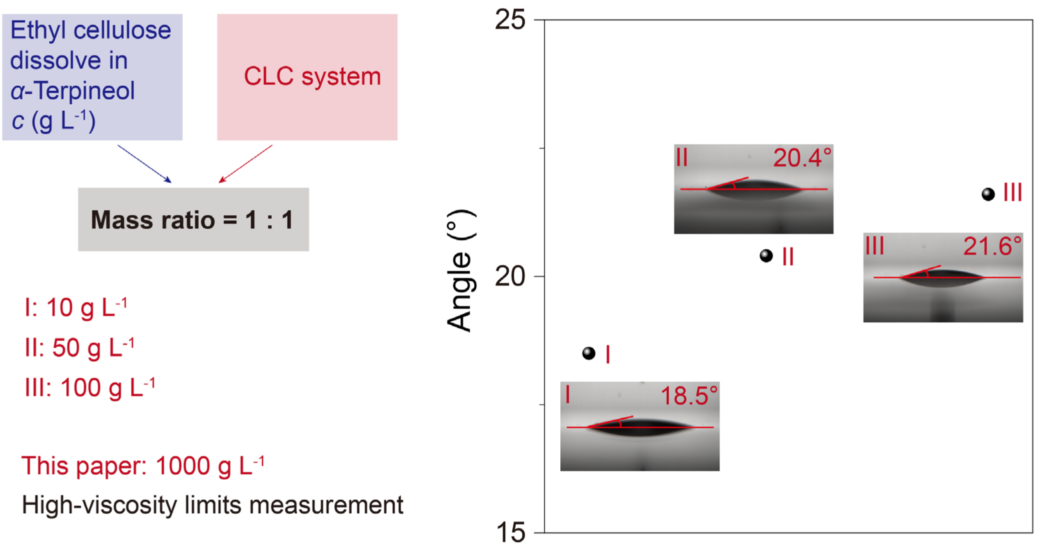

Fig. S2 Contact angles of paintable CLC system at different concentrations, including 10, 50, and 100 g L^{-1} ethyl cellulose in α-terpineol.

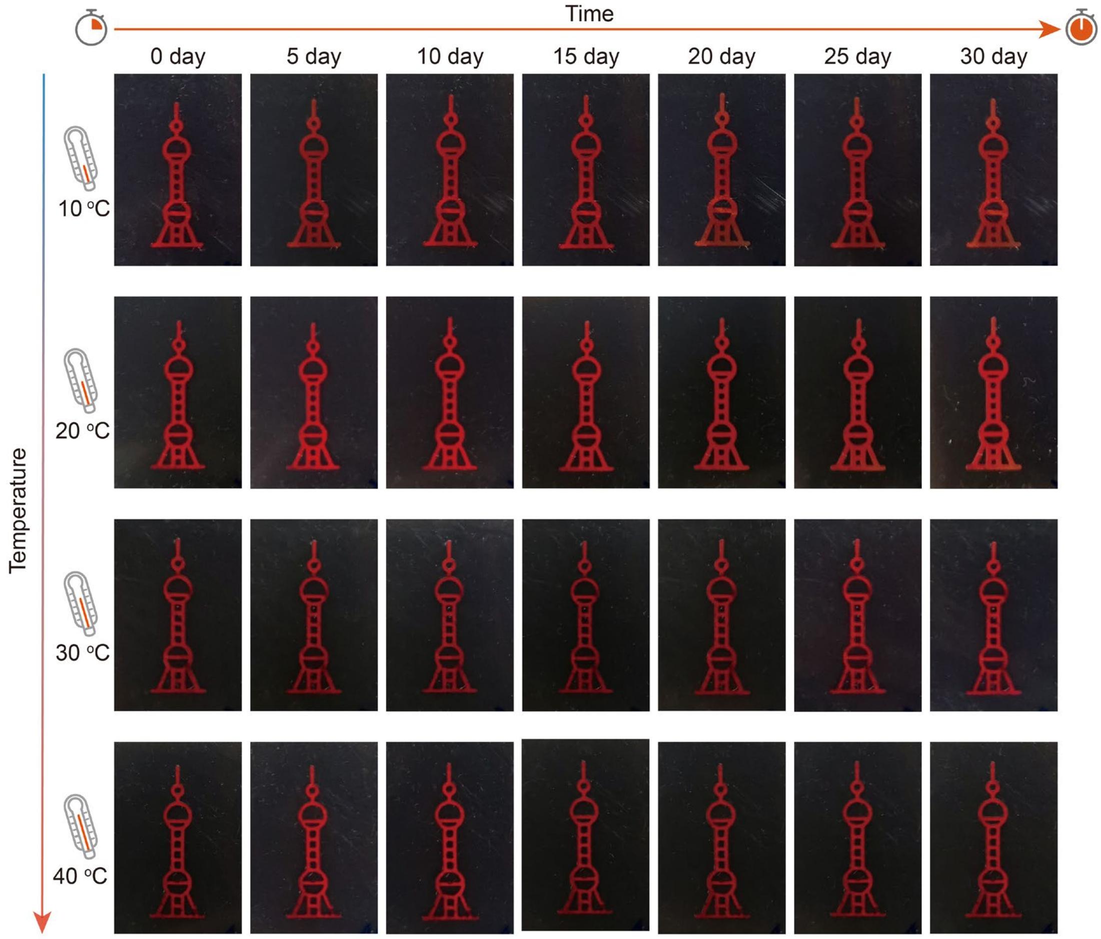

Fig. S3 Paintable optical superstructures exhibit outstanding thermal stability with unaffected color after exposure to various temperatures for 30 days.

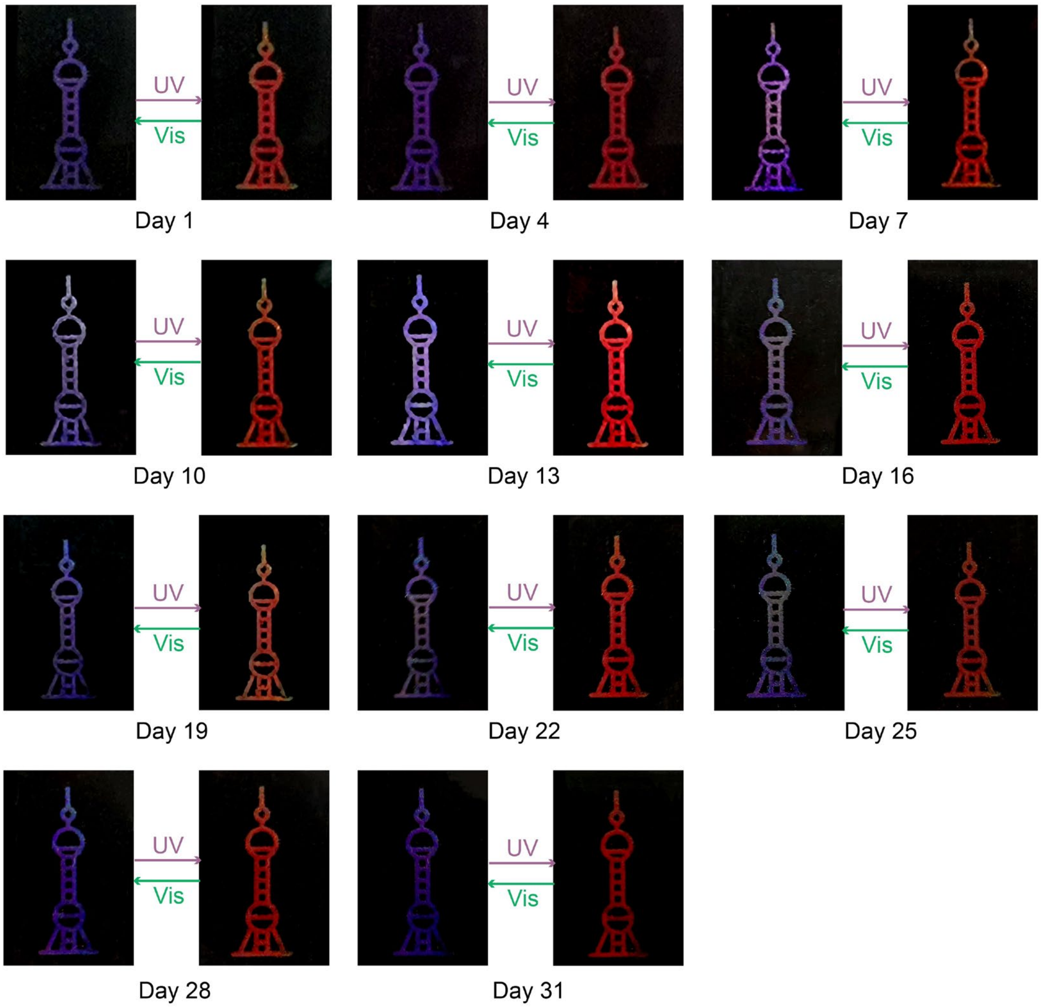

Fig. S4 Paintable optical superstructures exhibit outstanding thermal stability with unaffected color variation after exposure to -20 °C for 31 days.

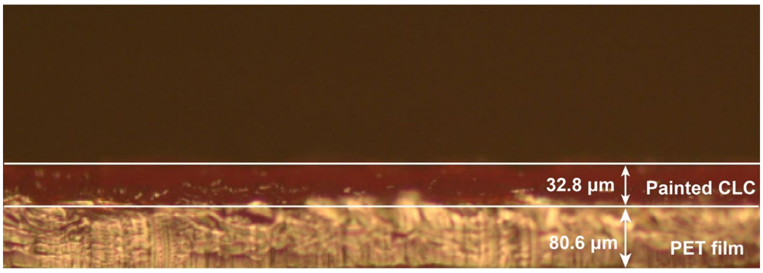

Fig. S5 Thickness of both the painted CLC and PET film.

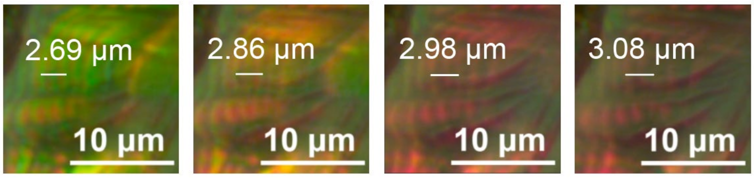

Fig. S6 Cross-sectional diagram of paintable optical superstructures controlled by light.

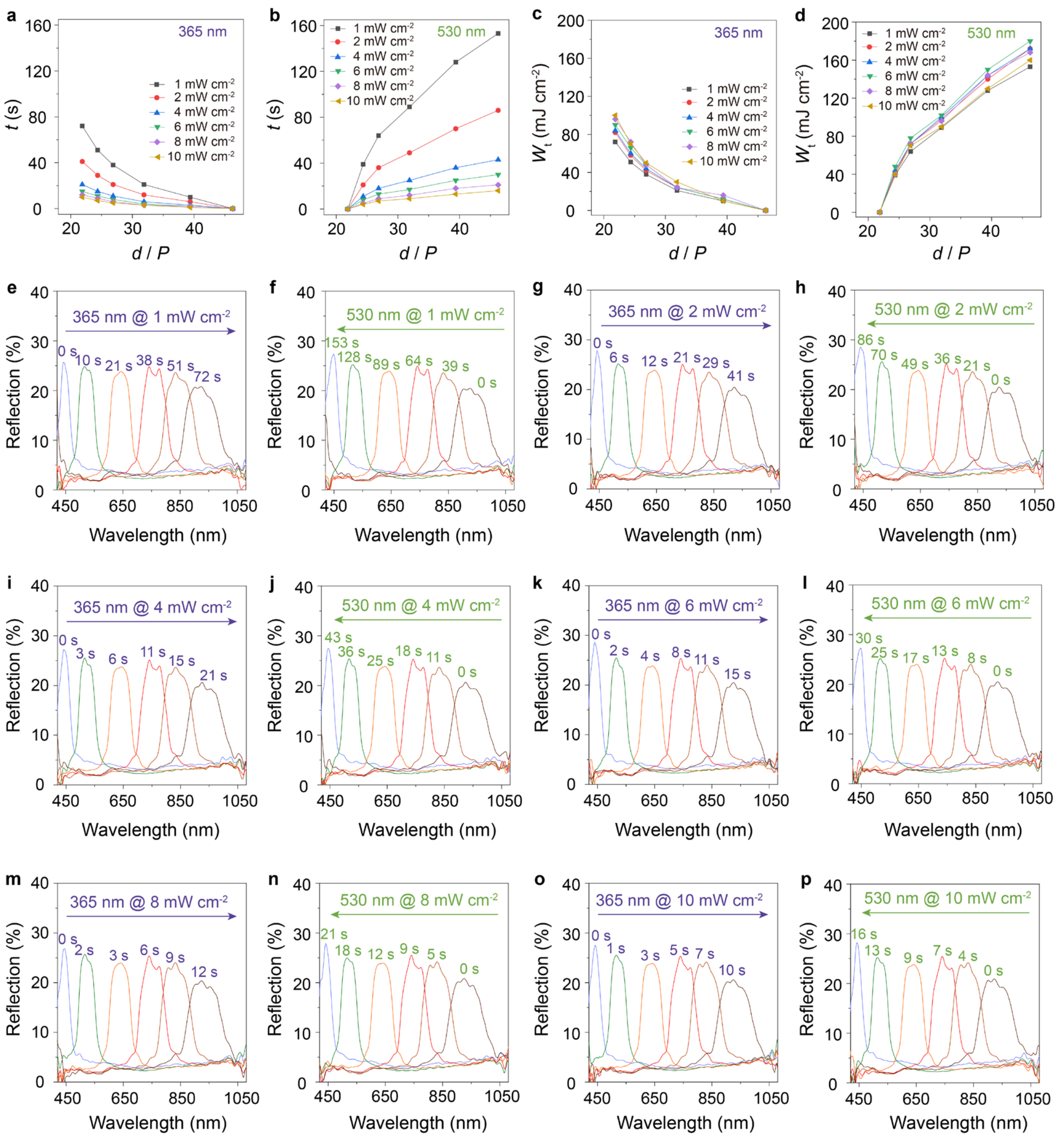

Fig. S7 The relationship between the film thickness (d), the chiral pitch (P), the irradiation time (t), and the light energy (W_t). **a,b** Relationship between time and thickness-to-pitch ratio under different power levels. **c,d** Relationship between light energy and thickness-to-pitch ratio under different power levels. **e-p** Reflection spectra of paintable CLC under different power levels. Here, film thickness d = 32.8 μm, CLC effective refractive index n = 1.6.

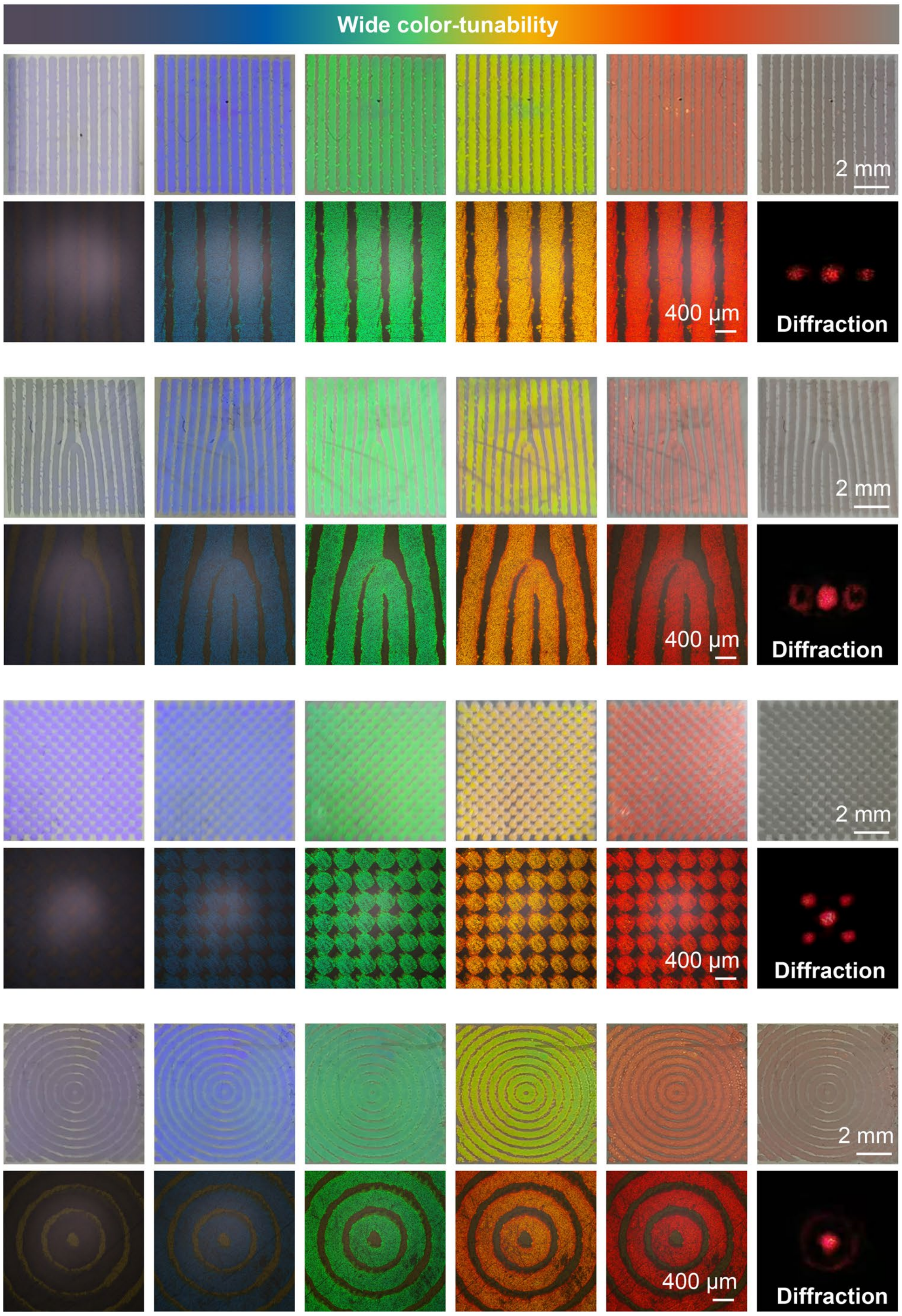

Fig. S8 Paintable optical micropatterns of one-dimension grating, fork-shaped grating, two-dimension grating, and periodic concentric circles with dark purple, blue, green, yellow, and red reflection colors were generated using a 365 nm UV light.

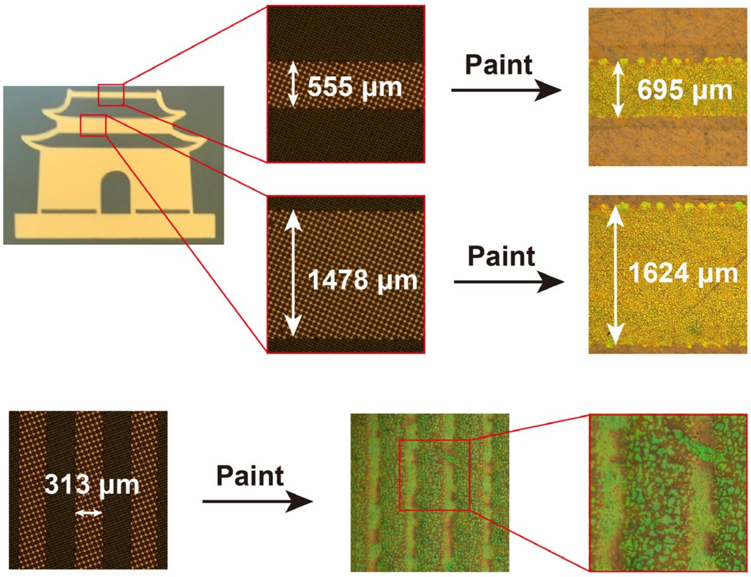

Fig. S9 Paintable photonic superstructures at different resolution.

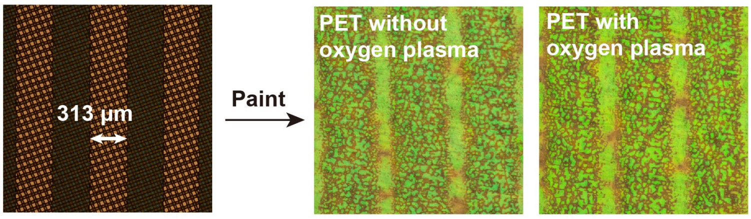

Fig. S10 Paintable photonic superstructures at PET film without/with oxygen plasma treatment.

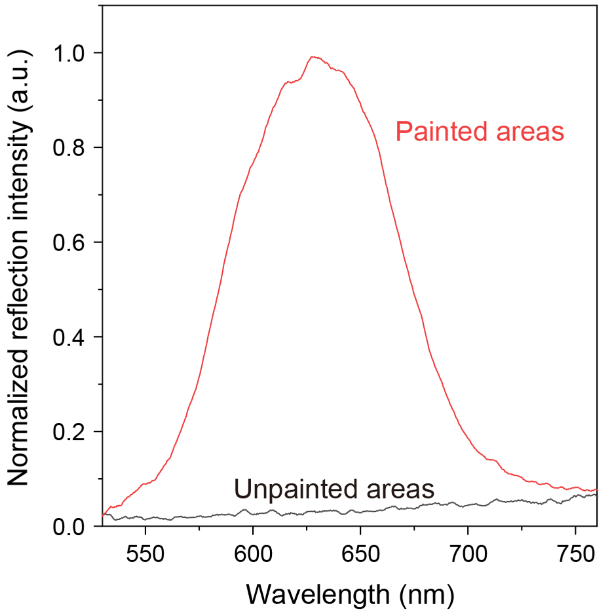

Fig. S11 Normalized reflection intensity of the painted and unpainted photoresponsive CLC areas.

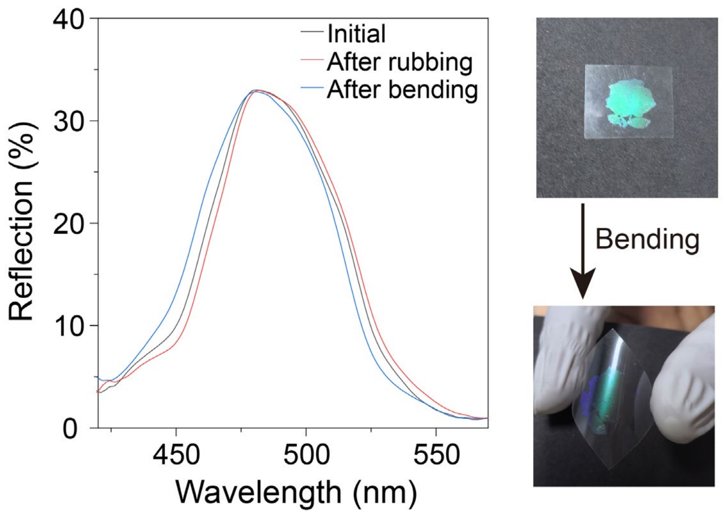

Fig. S12 Reflection spectra of paintable CLC with the bending and rubbing process.

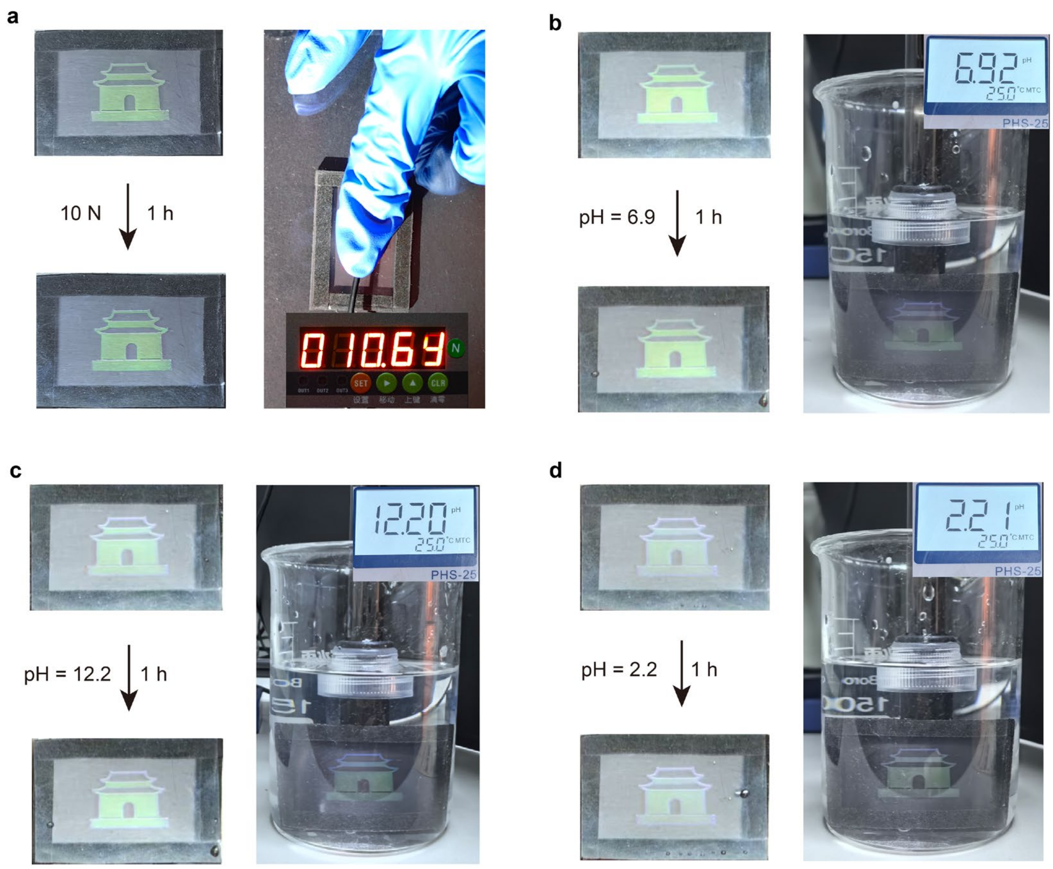

Fig. S13 stability tests under varying conditions, including **a)** pressure levels, **b)** water, **c)** acidic, and **d)** alkaline solutions.

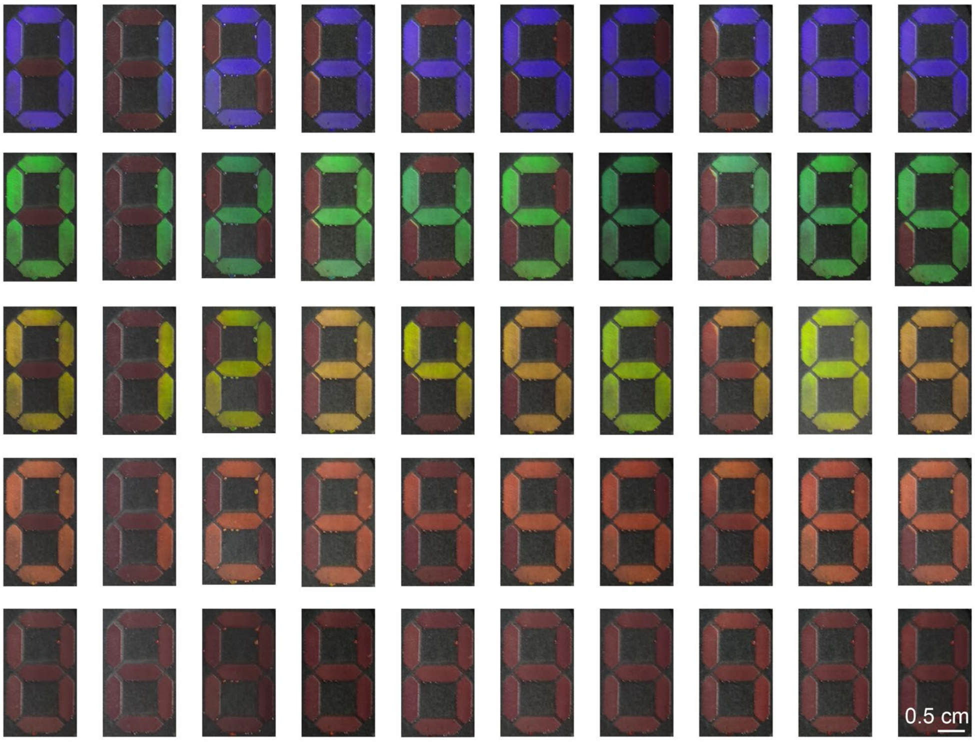

Fig. S14 Colorful display device of 0-9 numbers is achieved by illuminating different regions.

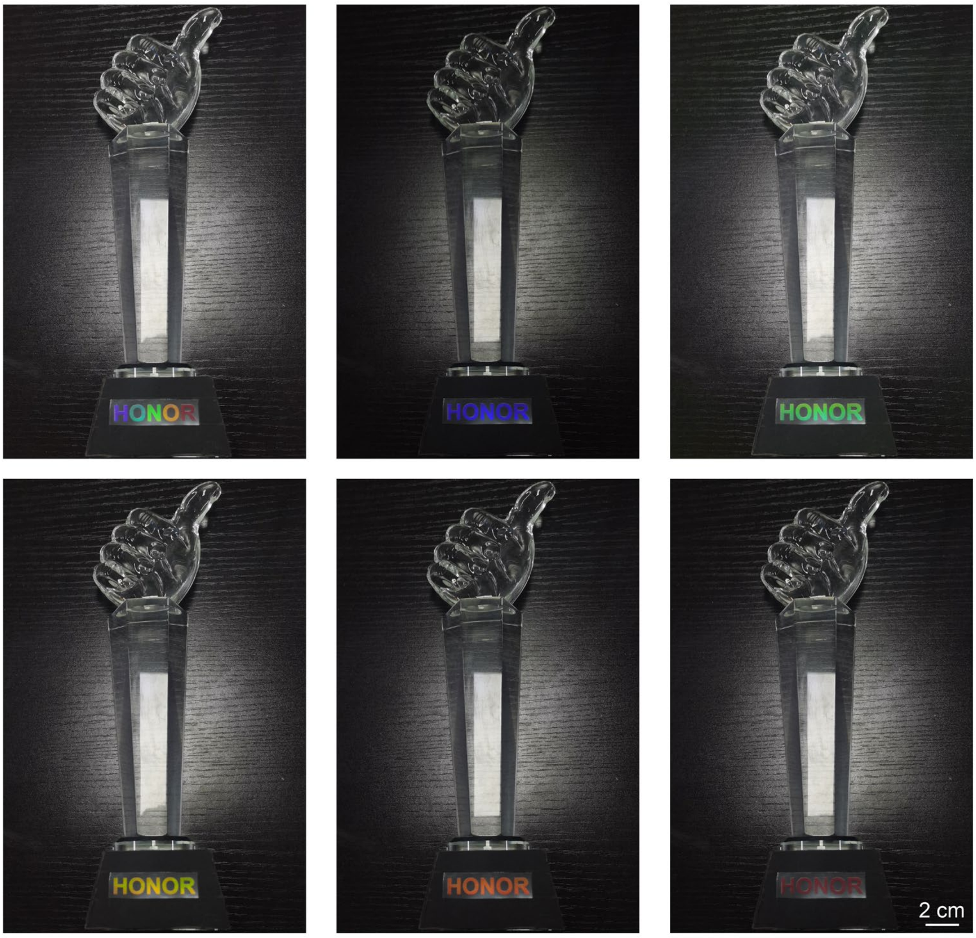

Fig. S15 Colorful “HONOR” on the trophy is achieved in promising applications.

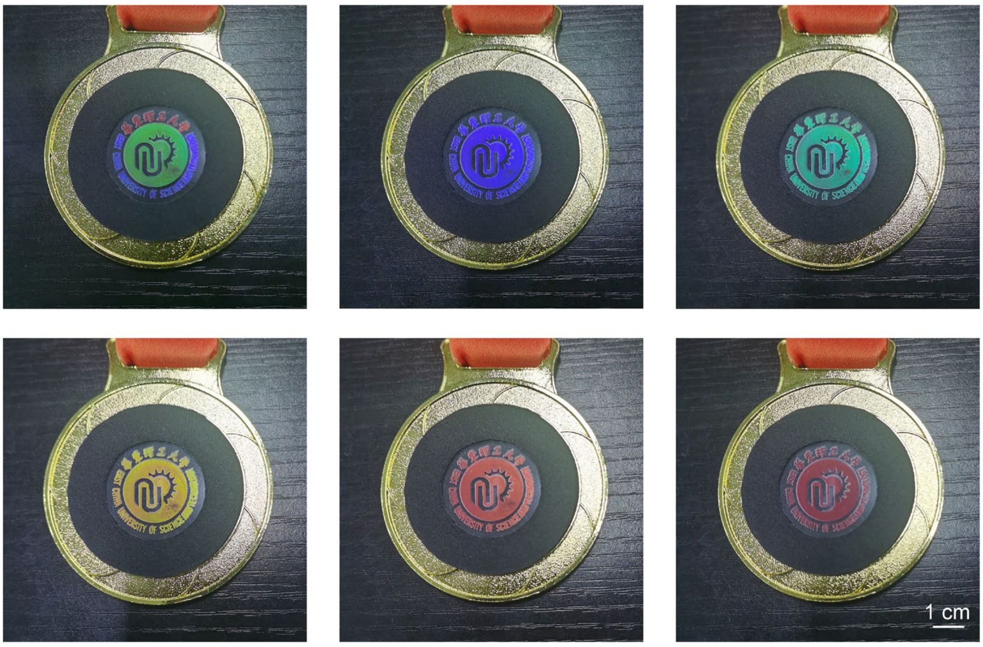

Fig. S16 Colorful "ECUST logo" on the medal is achieved in promising applications.

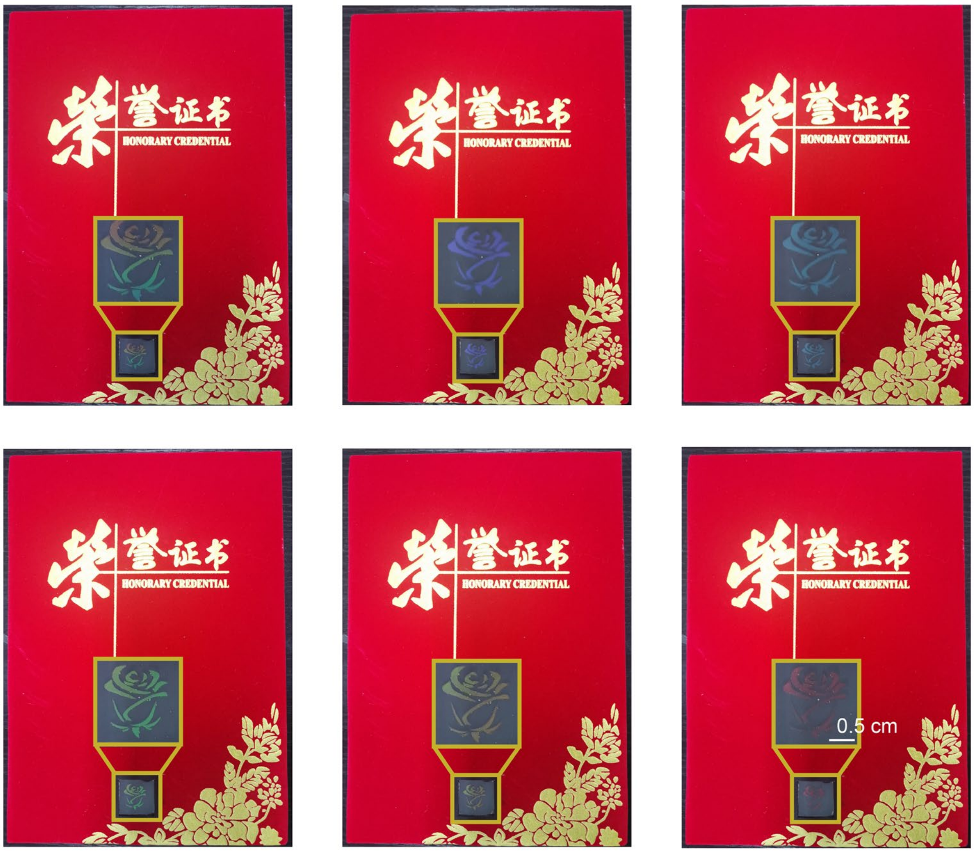

Fig. S17 Colorful "rose" on the certificates is achieved in promising applications.

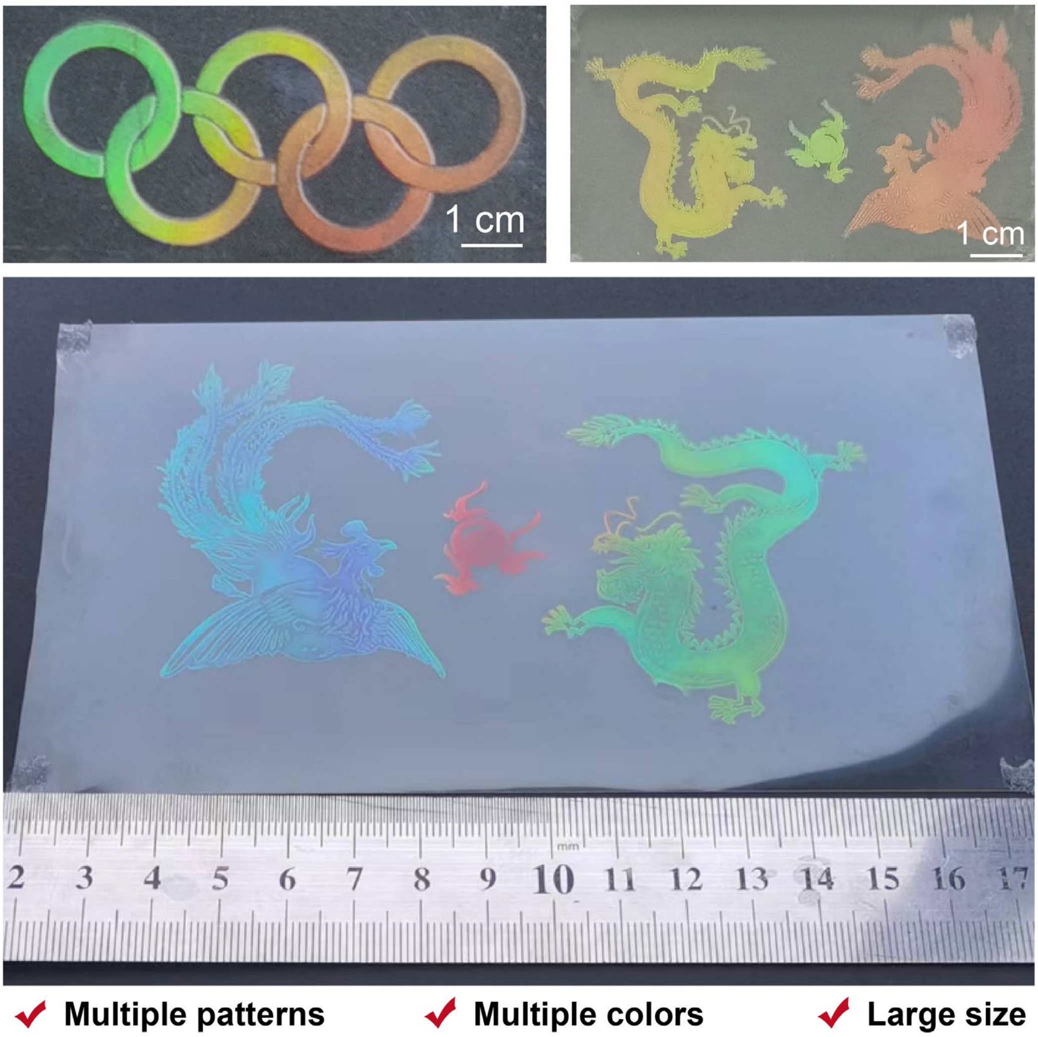

Fig. S18 The paintable optical superstructures can be adaptable to multiple patterns, colors, and large size.

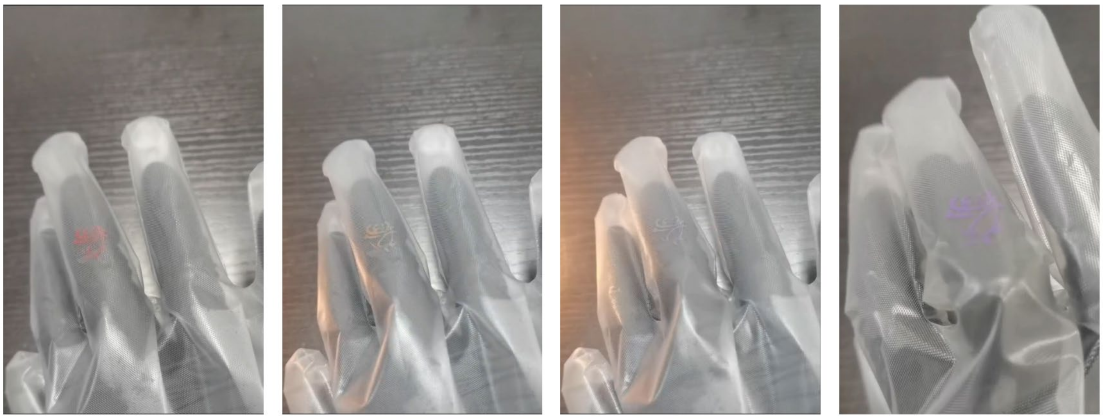

Fig. S19 The paintable optical superstructures can be adaptable to wearable multicolor devices.

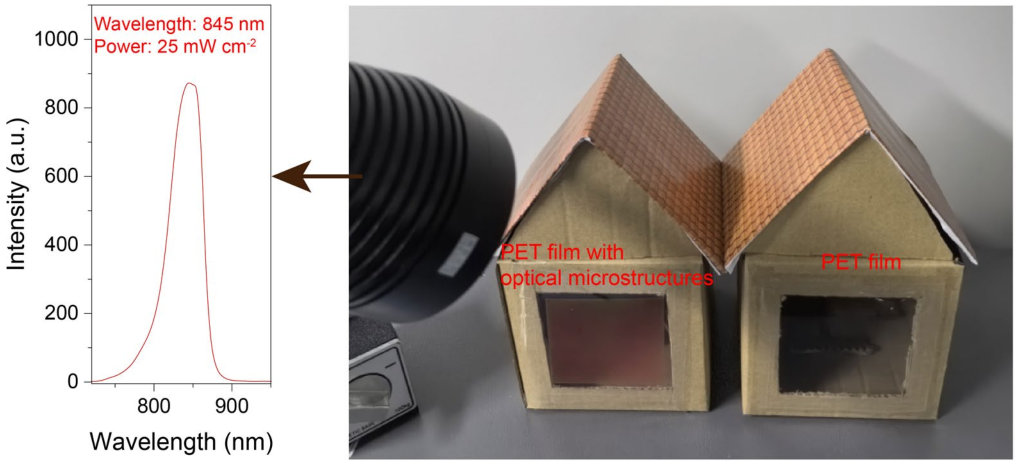

Fig. S20 Smart window film performance testing using NIR light (845 nm, 25 mW cm^{-2}): Comparative analysis of films with and without optical microstructures.

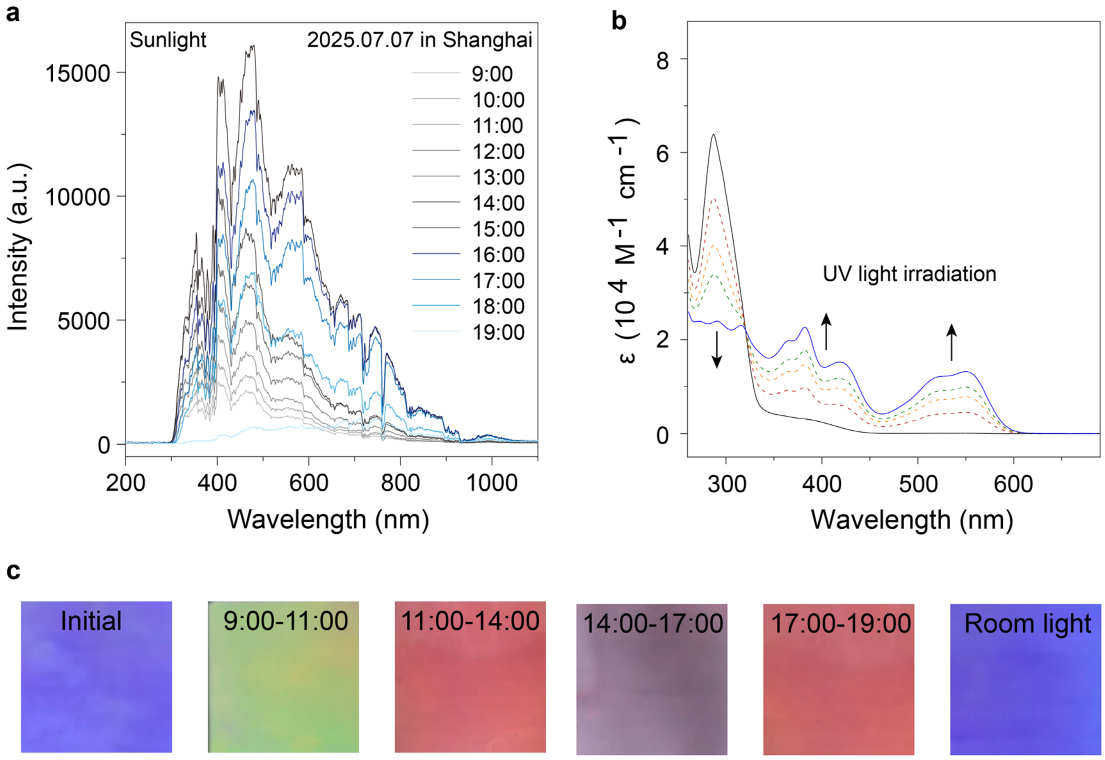

Fig. S21 Performance of smart window film in natural environments. **a** Full-spectrum solar irradiance recorded in Shanghai (07/07/2025). **b** Absorption spectra of the photoswitch. **c** Time-dependent reflection color changes of the smart window film.
